# The genome sequence of the iron prominent, *Notodonta dromedarius *(Linnaeus, 1767)

**DOI:** 10.12688/wellcomeopenres.17489.1

**Published:** 2021-12-14

**Authors:** Douglas Boyes, Peter W.H. Holland, University of Oxford and Wytham Woods Genome Acquisition Lab, Darwin Tree of Life Barcoding collective, Wellcome Sanger Institute Tree of Life programme, Wellcome Sanger Institute Scientific Operations: DNA Pipelines collective, Tree of Life Core Informatics collective, Darwin Tree of Life Consortium

**Affiliations:** 1 UK Centre for Ecology and Hydrology, Wallingford, Oxfordhsire, UK; 2 Department of Zoology, University of Oxford, Oxford, UK

**Keywords:** Notodonta dromedarius, iron prominent, genome sequence, chromosomal, Lepidoptera

## Abstract

We present a genome assembly from an individual male *Notodonta dromedarius* (iron prominent; Arthropoda; Insecta; Lepidoptera; Notodontidae). The genome sequence is 342 megabases in span. The majority of the assembly, 99.35%, is scaffolded into 31 chromosomal pseudomolecules, with the Z sex chromosome assembled.

## Species taxonomy

Eukaryota; Metazoa; Ecdysozoa; Arthropoda; Hexapoda; Insecta; Pterygota; Neoptera; Endopterygota; Lepidoptera; Glossata; Ditrysia; Noctuoidea; Notodontidae; Notodontinae; Notodonta; *Notodonta dromedarius* (Linnaeus, 1767) (NCBI:txid753204).

## Background

*Notodonta dromedarius* (iron prominent) has rust-coloured wing markings that give the moth its common name. The species is widely distributed across Europe and is common throughout the UK; however, abundance has greatly decreased at monitored sites over the past 50 years ( Randle *et al.*, 2019). There are two broods of *N. dromedarius* in the south of England flying in May/June and August, but usually a single brood in the north of England and in Scotland ( Randle *et al.*, 2019). The moth was one of the first members of the Notodontidae to have the sex pheromone chemical identified ( Bestmann *et al.*, 1993). The genome of *N. dromedarius* was sequenced as part of the Darwin Tree of Life Project, a collaborative effort to sequence all of the named eukaryotic species in the Atlantic Archipelago of Britain and Ireland. Here we present a chromosomally complete genome sequence for *N. dromedarius*, based on one male specimen from Wytham Woods, Oxfordshire, UK.

## Genome sequence report

**Figure 1.**
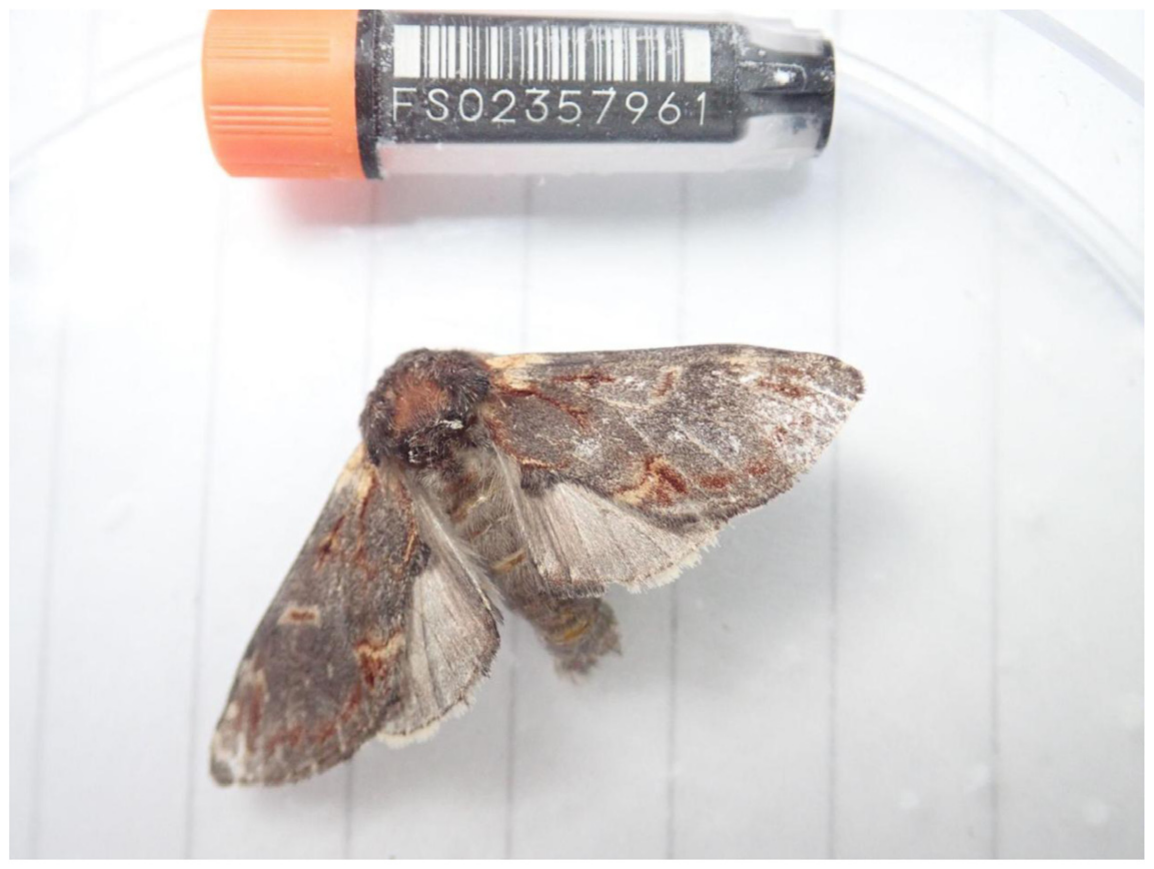
Image of the ilNotDrom1 specimen taken prior to preservation and processing. Specimen shown next to FluidX storage tube, 43.9 mm in length.

The genome was sequenced from a single male *N. dromedarius* ( Figure 1) collected from Wytham Woods, Oxfordshire, UK (latitude 51.772, longitude -1.338). A total of 77-fold coverage in Pacific Biosciences single-molecule long reads (N50 13 kb) and 112-fold coverage in 10X Genomics read clouds were generated. Primary assembly contigs were scaffolded with chromosome conformation Hi-C data. Manual assembly curation corrected 6 missing/misjoins and removed 56 haplotypic duplications, reducing the assembly length by 0.83% and the scaffold number by 28.57%, and increasing the scaffold N50 by 3.08%.

**Figure 2.**
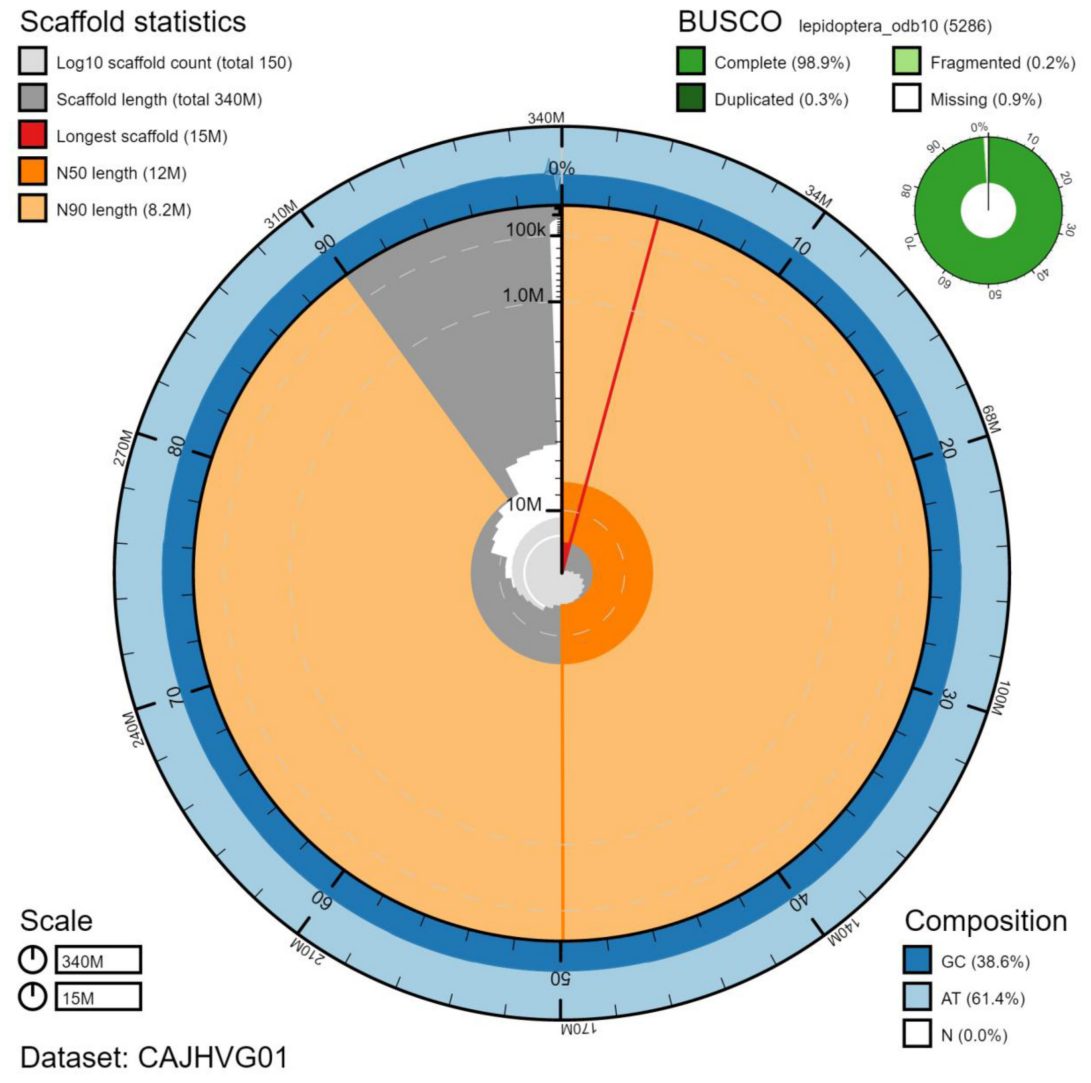
Genome assembly of *Notodonta dromedarius*, ilNotDrom1.1: metrics. The BlobToolKit Snailplot shows N50 metrics and BUSCO gene completeness. The main plot is divided into 1,000 size-ordered bins around the circumference with each bin representing 0.1% of the 341,992,784 bp assembly. The distribution of chromosome lengths is shown in dark grey with the plot radius scaled to the longest chromosome present in the assembly (14,515,539 bp, shown in red). Orange and pale-orange arcs show the N50 and N90 chromosome lengths (12,059,830 and 8,218,830 bp), respectively. The pale grey spiral shows the cumulative chromosome count on a log scale with white scale lines showing successive orders of magnitude. The blue and pale-blue area around the outside of the plot shows the distribution of GC, AT and N percentages in the same bins as the inner plot. A summary of complete, fragmented, duplicated and missing BUSCO genes in the lepidoptera_odb10 set is shown in the top right. An interactive version of this figure is available at https://blobtoolkit.genomehubs.org/view/ilNotDrom1.1/dataset/CAJHVG01/snail.

The final assembly has a total length of 342 Mb in 145 sequence scaffolds with a scaffold N50 of 12.1 Mb ( Table 1). Of the assembly sequence, 99.35% was assigned to 31 chromosomal-level scaffolds, representing 30 autosomes (numbered by sequence length), and the Z sex chromosome ( Figure 2– Figure 5; Table 2). The assembly has a BUSCO v5.1.2 ( Simão *et al.*, 2015) completeness of 98.9% (single 98.6%, duplicated 0.3%) using the lepidoptera_odb10 reference set. While not fully phased, the assembly deposited is of one haplotype. Contigs corresponding to the second haplotype have also been deposited.

**Table 1.** Genome data for *Notodonta dromedarius*, ilNotDrom1.1.

*Project accession data*	
Assembly identifier	ilNotDrom1.1
Species	*Notodonta dromedarius*
Specimen	ilNotDrom1
NCBI taxonomy ID	NCBI:txid753204
BioProject	PRJEB42138
BioSample ID	SAMEA7520190
Isolate information	Male, head/thorax, abdomen
*Raw data accessions*	
PacificBiosciences SEQUEL II	ERR6590583
10X Genomics Illumina	ERR6002703-ERR6002706
Hi-C Illumina	ERR6003044
Illumina PolyA RNA-Seq	ERR6286708
*Genome assembly*	
Assembly accession	GCA_905147325.1
*Accession of alternate haplotype*	GCA_905147855.1
Span (Mb)	342
Number of contigs	168
Contig N50 length (Mb)	10
Number of scaffolds	146
Scaffold N50 length (Mb)	12
Longest scaffold (Mb)	15
BUSCO * genome score	C:98.9%[S:98.6%,D:0.3%],F:0.2%,M:0.9%,n:5286

*BUSCO scores based on the lepidoptera_odb10 BUSCO set using v5.1.2. C= complete [S= single copy, D=duplicated], F=fragmented, M=missing, n=number of orthologues in comparison. A full set of BUSCO scores is available at https://blobtoolkit.genomehubs.org/view/ilNotDrom1.1/dataset/CAJHVG01/busco.

**Figure 3.**
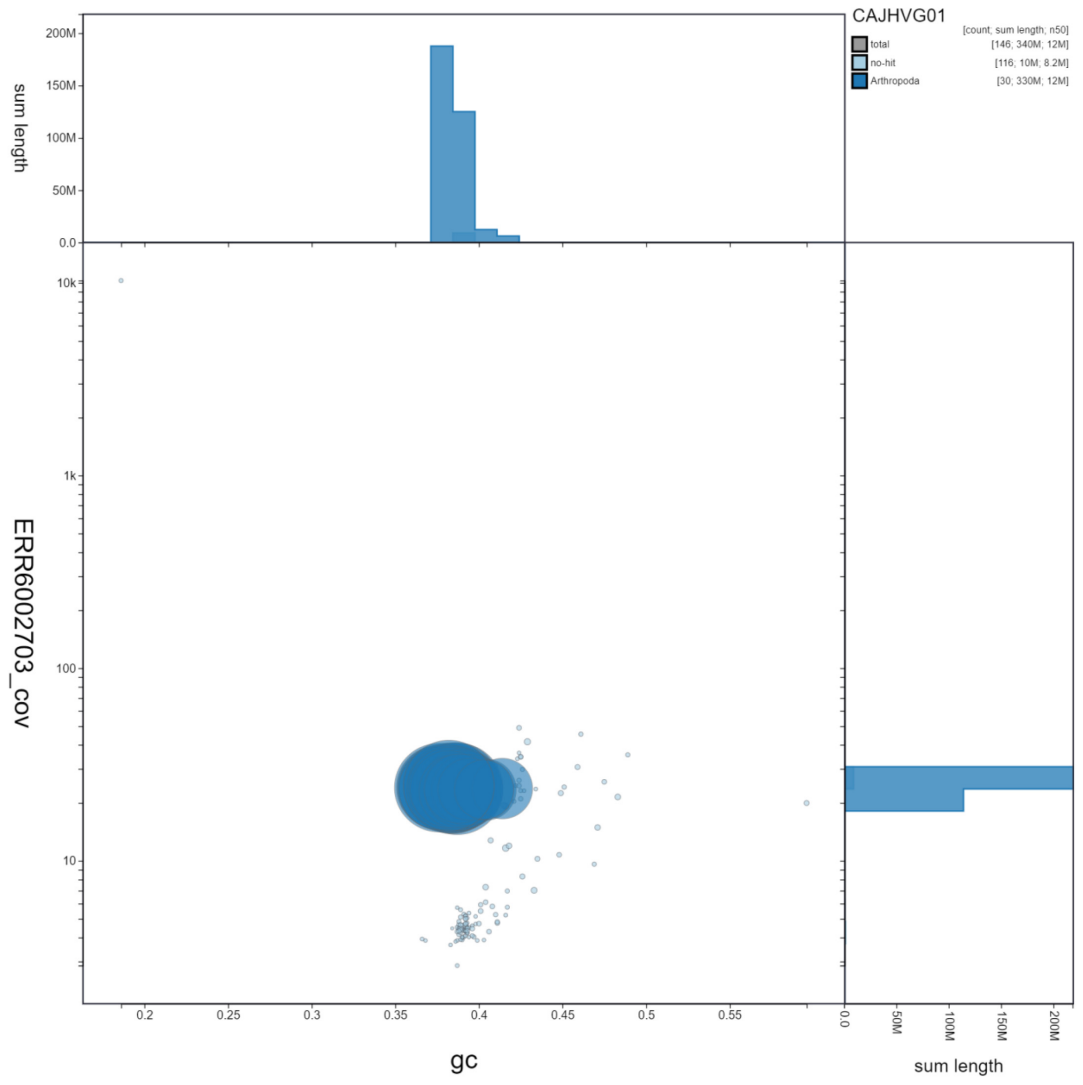
Genome assembly of *Notodonta dromedarius*, ilNotDrom1.1: GC coverage. BlobToolKit GC-coverage plot. Scaffolds are coloured by phylum. Circles are sized in proportion to scaffold length. Histograms show the distribution of scaffold length sum along each axis. An interactive version of this figure is available at https://blobtoolkit.genomehubs.org/view/ilNotDrom1.1/dataset/CAJHVG01/blob.

**Figure 4.**
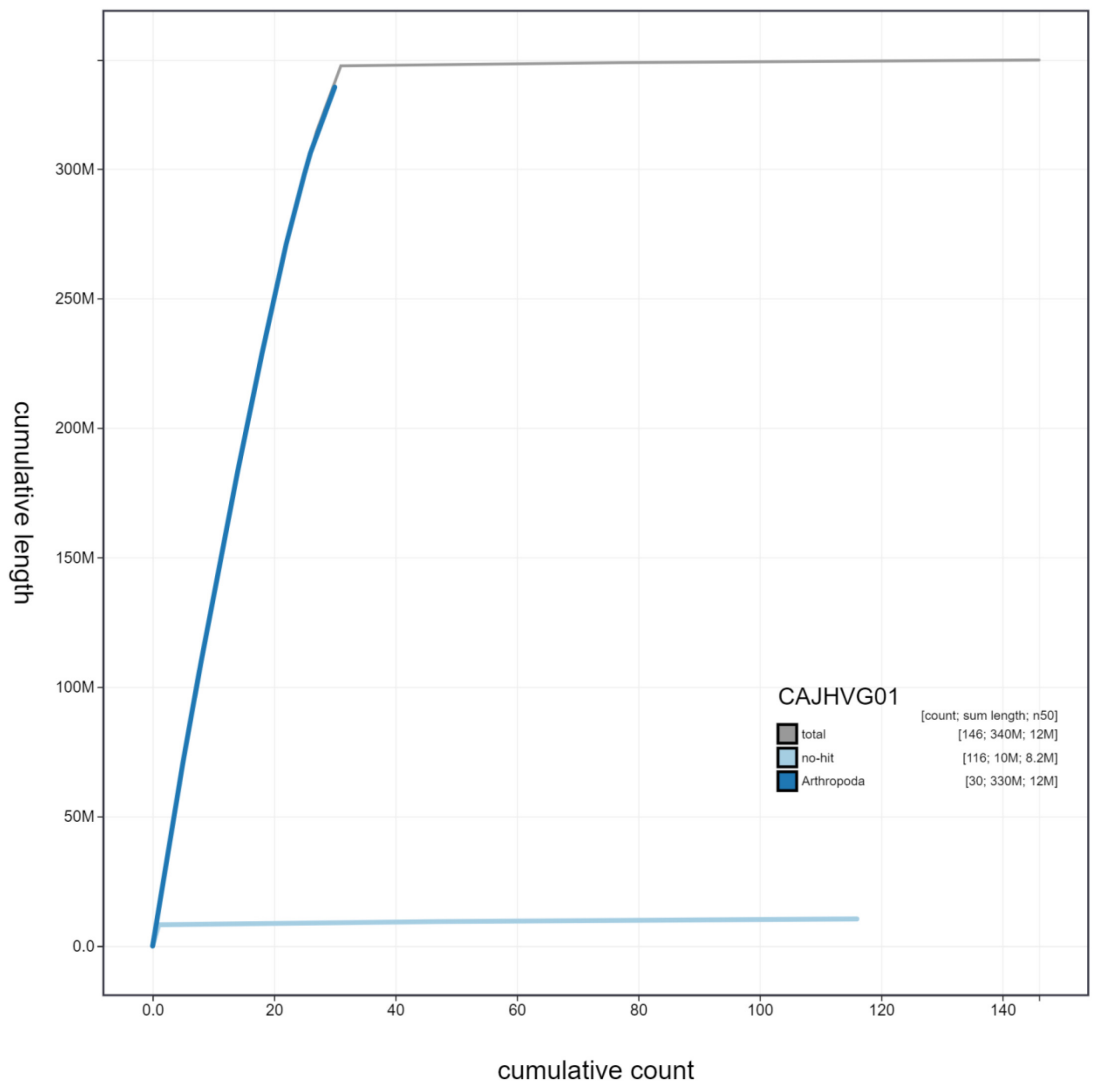
Genome assembly of *Notodonta dromedarius*, ilNotDrom1.1: cumulative sequence. BlobToolKit cumulative sequence plot. The grey line shows cumulative length for all scaffolds. Coloured lines show cumulative lengths of scaffolds assigned to each phylum using the buscogenes taxrule. An interactive version of this figure is available at https://blobtoolkit.genomehubs.org/view/ilNotDrom1.1/dataset/CAJHVG01/cumulative.

**Figure 5.**
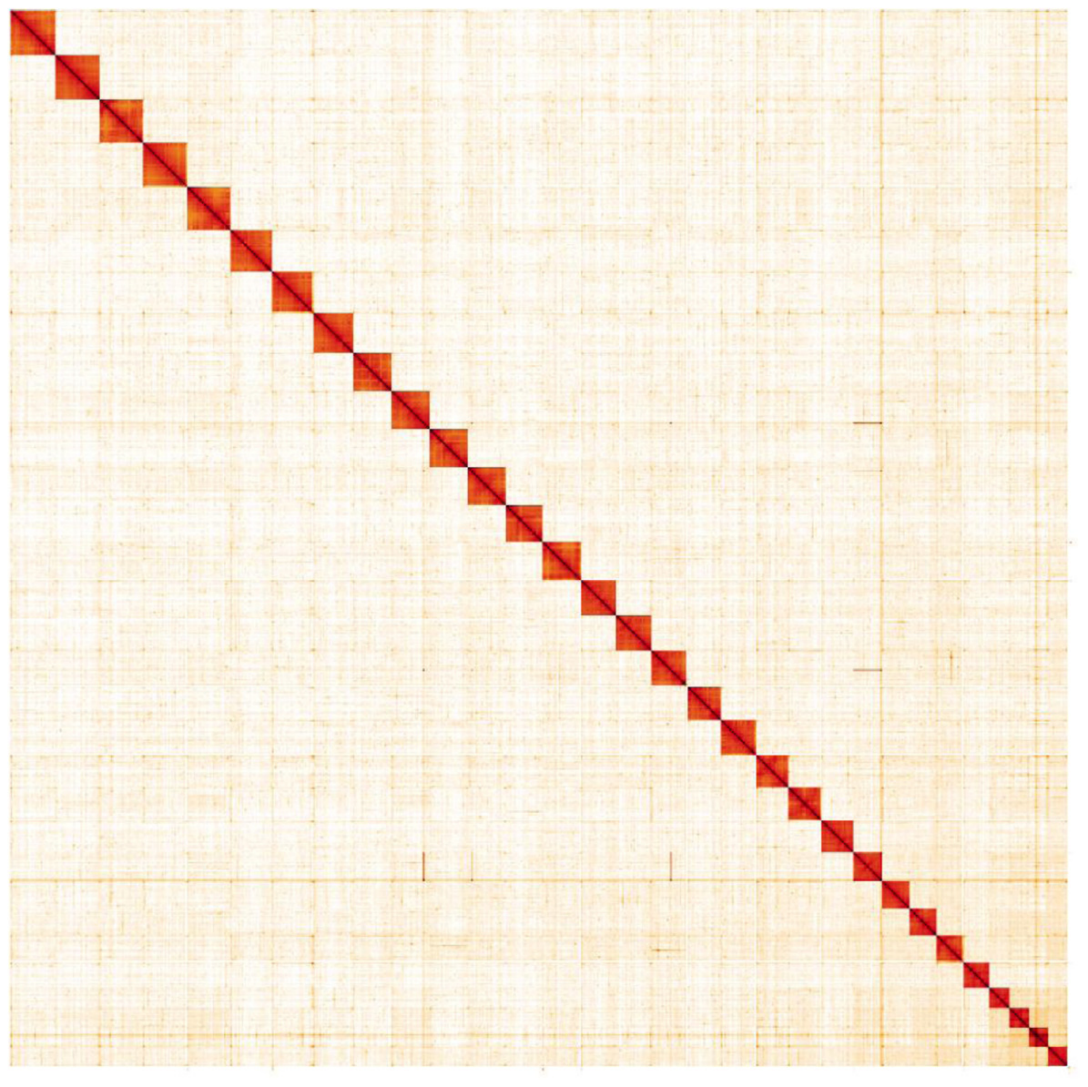
Genome assembly of *Notodonta dromedarius*, ilNotDrom1.1: Hi-C contact map. Hi-C contact map of the ilNotDrom1.1 assembly, visualised in HiGlass. Chromosomes are shown in order of size from left to right and top to bottom.

**Table 2.** Chromosomal pseudomolecules in the genome assembly of *Notodonta dromedarius*, ilNotDrom1.1.

INSDC accession	Chromosome	Size (Mb)	GC%
LR990159.1	1	14.52	38.7
LR990161.1	2	14.15	38.4
LR990162.1	3	14.00	38.2
LR990163.1	4	13.87	38.6
LR990164.1	5	13.54	37.6
LR990165.1	6	13.03	38.4
LR990166.1	7	12.72	38.4
LR990167.1	8	12.46	37.7
LR990168.1	9	12.33	38.3
LR990169.1	10	12.21	37.6
LR990170.1	11	12.13	38.4
LR990171.1	12	12.06	37.7
LR990172.1	13	11.99	38.2
LR990173.1	14	11.70	38.7
LR990174.1	15	11.32	38.1
LR990175.1	16	11.28	38.7
LR990176.1	17	11.13	38.2
LR990177.1	18	10.91	39
LR990178.1	19	10.72	38.8
LR990179.1	20	10.34	39.1
LR990180.1	21	10.30	38.2
LR990181.1	22	9.21	38.6
LR990182.1	23	8.99	38.9
LR990183.1	24	8.74	39.3
LR990184.1	25	8.29	38.8
LR990185.1	26	8.22	39.1
LR990186.1	27	6.60	39.4
LR990187.1	28	6.38	40.4
LR990188.1	29	6.25	41.4
LR990189.1	30	6.08	40.3
LR990160.1	Z	14.29	38.2
LR990190.1	MT	0.02	19.2
-	Unplaced	2.22	41.3

## Methods

### Sample acquisition and nucleic acid extraction

A single male *N. dromedarius* (ilNotDrom1) was collected from Wytham Woods, Oxfordshire, UK (latitude 51.772, longitude -1.338) by Douglas Boyes, UKCEH, using a light trap. The specimen was identified by the same individual and preserved on dry ice.

DNA was extracted from head/thorax tissue at the Wellcome Sanger Institute (WSI) Scientific Operations core from the whole organism using the Qiagen MagAttract HMW DNA kit, according to the manufacturer’s instructions. RNA was extracted (also from head/thorax tissue) in the Tree of Life Laboratory at the WSI using TRIzol (Invitrogen), according to the manufacturer’s instructions. RNA was then eluted in 50 μl RNAse-free water and its concentration RNA assessed using a Nanodrop spectrophotometer and Qubit Fluorometer using the Qubit RNA Broad-Range (BR) Assay kit. Analysis of the integrity of the RNA was done using Agilent RNA 6000 Pico Kit and Eukaryotic Total RNA assay.

### Sequencing

Pacific Biosciences HiFi circular consensus and 10X Genomics read cloud DNA sequencing libraries, in addition to PolyA RNA-Seq libraries, were constructed according to the manufacturers’ instructions. DNA and RNA sequencing was performed by the Scientific Operations core at the WSI on Pacific Biosciences SEQUEL II (HiFi), Illumina HiSeq X (10X) and Illumina HiSeq 4000 (RNA-Seq) instruments. Hi-C data were generated from abdomen tissue of the same specimen using the Arima v1 Hi-C kit and sequenced on HiSeq X.

### Genome assembly

Assembly was carried out with HiCanu ( Nurk *et al.*, 2020); haplotypic duplication was identified and removed with purge_dups ( Guan *et al.*, 2020). One round of polishing was performed by aligning 10X Genomics read data to the assembly with longranger align, calling variants with freebayes ( Garrison & Marth, 2012). The assembly was then scaffolded with Hi-C data ( Rao *et al.*, 2014) using SALSA2 ( Ghurye *et al.*, 2019). The assembly was checked for contamination and corrected using the gEVAL system ( Chow *et al.*, 2016) as described previously ( Howe *et al.*, 2021). Manual curation ( Howe *et al.*, 2021) was performed using gEVAL, HiGlass ( Kerpedjiev *et al.*, 2018) and Pretext. The mitochondrial genome was assembled using MitoHiFi ( Uliano-Silva *et al.*, 2021) and annotated using MitoFinder ( Allio *et al.*, 2020). The genome was analysed and BUSCO scores generated within the BlobToolKit environment ( Challis *et al.*, 2020). Table 3 contains a list of all software tool versions used, where appropriate.

**Table 3.** Software tools used.

Software tool	Version	Source
HiCanu	1.0	Nurk *et al.*, 2020
purge_dups	1.2.3	Guan *et al.*, 2020
SALSA2	2.2	Ghurye *et al.*, 2019
longranger align	2.2.2	https://support.10xgenomics.com/genome-exome/software/pipelines/latest/advanced/other-pipelines
freebayes	1.3.1-17-gaa2ace8	Garrison & Marth, 2012
gEVAL	N/A	Chow *et al.*, 2016
HiGlass	1.11.6	Kerpedjiev *et al.*, 2018
PretextView	0.1.x	https://github.com/wtsi-hpag/PretextView
BlobToolKit	2.6.2	Challis *et al.*, 2020

### Ethics/compliance issues

The materials that have contributed to this genome note have been supplied by a Darwin Tree of Life Partner. The submission of materials by a Darwin Tree of Life Partner is subject to the Darwin Tree of Life Project Sampling Code of Practice. By agreeing with and signing up to the Sampling Code of Practice, the Darwin Tree of Life Partner agrees they will meet the legal and ethical requirements and standards set out within this document in respect of all samples acquired for, and supplied to, the Darwin Tree of Life Project. Each transfer of samples is further undertaken according to a Research Collaboration Agreement or Material Transfer Agreement entered into by the Darwin Tree of Life Partner, Genome Research Limited (operating as the Wellcome Sanger Institute), and in some circumstances other Darwin Tree of Life collaborators.

## Data availability

European Nucleotide Archive: Notodonta dromedarius (iron prominent). Accession number PRJEB42138: https://www.ebi.ac.uk/ena/browser/view/PRJEB42138.

The genome sequence is released openly for reuse. The *N. dromedarius* genome sequencing initiative is part of the Darwin Tree of Life (DToL) project. All raw sequence data and the assembly have been deposited in INSDC databases. The genome will be annotated using the RNA-Seq data and presented through the Ensembl pipeline at the European Bioinformatics Institute. Raw data and assembly accession identifiers are reported in Table 1.

## Author information

Members of the University of Oxford and Wytham Woods Genome Acquisition Lab are listed here: https://doi.org/10.5281/zenodo.5746938.

Members of the Darwin Tree of Life Barcoding collective are listed here: https://doi.org/10.5281/zenodo.5744972.

Members of the Wellcome Sanger Institute Tree of Life programme are listed here: https://doi.org/10.5281/zenodo.5744840.

Members of Wellcome Sanger Institute Scientific Operations: DNA Pipelines collective are listed here: https://doi.org/10.5281/zenodo.5746904.

Members of the Tree of Life Core Informatics collective are listed here: https://doi.org/10.5281/zenodo.5743293.

Members of the Darwin Tree of Life Consortium are listed here: https://doi.org/10.5281/zenodo.5638618.
