# Supplementary material for: The genome sequence of the iron prominent, Notodonta dromedarius (Linnaeus, 1767)
Source: Wellcome Open Res. 2021 Dec 14;6:341. [Version 1] doi: 10.12688/wellcomeopenres.17489.1 (PMC11190643; doi:10.12688/wellcomeopenres.17489.1)
Supplement: Peer review history

# Reviewer report: Reviewer response for version 1

**DOI:** 10.21956/wellcomeopenres.19339.r83095

**Published:** 2024-06-20

**Authors:** Camille Cornet

**Affiliations:** 1 University of Neuchatel, Neuchâtel, Switzerland

The authors present a reference genome assembly for the moth species *Notodonta dromedarius*. The methods used to sequence and assemble the genome are sound and well reported in general. The resulting assembly is of high quality. This resource is likely to be very useful for future studies, for example of population genetics of the species and comparative genomics of Lepidoptera. I have only a few comments that I hope might improve this genome note.

 Background section:

 It would be good to provide some information on the ecology of the species. For example, in which habitat can it be found, what is the host plant of the caterpillar, are adults pollinators?

 Methods:

 There is an incongruence in this sentence: “DNA was extracted from head/thorax tissue at the Wellcome Sanger Institute (WSI) Scientific Operations core from the whole organism”. Was DNA extracted from the whole organism or just the head and thorax?

 Hi-C sequencing yield (number of reads) should be mentioned. Ideally also the percentage of read pairs mapping to a different contig prior to scaffolding.

 Some steps in the genome assembly could be better explained, for example, how were the 31 chromosomes inferred from the 146 scaffolds? Was it just a matter of size? How was the Z chromosome identified? Simply using depth of coverage from the PacBio reads mapped back to the assembly?

 Most importantly, there is no mention of genome annotation (except a small note in the Data availability statement), while RNA sequencing is well described, and annotation is reported online as “complete”. An entire section about genome annotation should be added, including methods used, number of protein-coding genes annotated, etc.

Are sufficient details of methods and materials provided to allow replication by others?

Partly

Is the rationale for creating the dataset(s) clearly described?

Partly

Are the datasets clearly presented in a useable and accessible format?

Yes

Are the protocols appropriate and is the work technically sound?

Yes

Reviewer Expertise:

Comparative genomics, population genomics

I confirm that I have read this submission and believe that I have an appropriate level of expertise to confirm that it is of an acceptable scientific standard, however I have significant reservations, as outlined above.

# Reviewer report: Reviewer response for version 1

**DOI:** 10.21956/wellcomeopenres.19339.r83099

**Published:** 2024-06-08

**Authors:** Luc Swevers

**Affiliations:** 1 National Center for Scientific Research Demokritos, Athens, Greece

More background information can be presented (ecology, habitat). Although very few articles can be found of the presented species, much more information exists with respect to the family of the Notodontidae and their characteristics (e.g. defense substances, coloration).

 In Table 1, the author of the species name can be mentioned.

 It can be explicitly stated that “iron prominent” is the common name.

 The system of sex chomosomes can be mentioned.

 Sequencing results are of high quality but can be explained better for the non-specialist.

 The purpose of RNA-seq can be explained.

 In Table 2, “MT” should be explained (mitochondria).

 In Figure 2, up to 31, each scaffold represents a chromosome (if this reviewer understands correctly). It can be mentioned that the sex chromosome represents the second largest scaffold.

Are sufficient details of methods and materials provided to allow replication by others?

Yes

Is the rationale for creating the dataset(s) clearly described?

Partly

Are the datasets clearly presented in a useable and accessible format?

Yes

Are the protocols appropriate and is the work technically sound?

Yes

Reviewer Expertise:

Insect molecular biology and biotechnology

I confirm that I have read this submission and believe that I have an appropriate level of expertise to confirm that it is of an acceptable scientific standard, however I have significant reservations, as outlined above.

# Reviewer report: Reviewer response for version 1

**DOI:** 10.21956/wellcomeopenres.19339.r83090

**Published:** 2024-05-22

**Authors:** Jason Hill

**Affiliations:** 1 Uppsala University, Uppsala, Sweden

In this study the authors describe the genome assembly and annotation of the European moth *Notodonta dromedarius*. The methods employed represent the current highest standard for assembly and annotation projects. This represents an additional excellent addition to the knowledge of the genomic architecture of Lepidoptera and will be useful for deeper studies of *Notodonta dromedarius* and for comparative genomics work in broader context.

Are sufficient details of methods and materials provided to allow replication by others?

Yes

Is the rationale for creating the dataset(s) clearly described?

Yes

Are the datasets clearly presented in a useable and accessible format?

Yes

Are the protocols appropriate and is the work technically sound?

Yes

Reviewer Expertise:

Population genomics, comparative genomics, Lepidoptera genetics

I confirm that I have read this submission and believe that I have an appropriate level of expertise to confirm that it is of an acceptable scientific standard.
